# Microwave-Assisted Desulfation of the Hemolytic Saponins Extracted from *Holothuria scabra* Viscera

**DOI:** 10.3390/molecules27020537

**Published:** 2022-01-15

**Authors:** Philippe Savarino, Emmanuel Colson, Guillaume Caulier, Igor Eeckhaut, Patrick Flammang, Pascal Gerbaux

**Affiliations:** 1 Organic Synthesis and Mass Spectrometry Laboratory (S²MOs), University of Mons, 23 Place du Parc, 7000 Mons, Belgium; philippe.savarino@umons.ac.be (P.S.); colson-emmanuel@outlook.be (E.C.); 2 Biology of Marine Organisms and Biomimetics Unit (BOMB), Research Institute for Biosciences, University of Mons, 23 Place du Parc, 7000 Mons, Belgium; guillaume.caulier@umons.ac.be (G.C.); igor.eeckhaut@umons.ac.be (I.E.); patrick.flammang@umons.ac.be (P.F.); 3 Belaza Marine Station, Institut Halieutique et des Sciences Marines (IH.SM), University of Toliara, Rue Dr Rabesandratana HD, Toliara 601, Madagascar

**Keywords:** saponins, triterpene glycosides, sea cucumbers, mass spectrometry, microwave activation, desulfation, hemolytic activity

## Abstract

Saponins are plant and marine animal specific metabolites that are commonly considered as molecular vectors for chemical defenses against unicellular and pluricellular organisms. Their toxicity is attributed to their membranolytic properties. Modifying the molecular structures of saponins by quantitative and selective chemical reactions is increasingly considered to tune the biological properties of these molecules (i) to prepare congeners with specific activities for biomedical applications and (ii) to afford experimental data related to their structure–activity relationship. In the present study, we focused on the sulfated saponins contained in the viscera of *Holothuria scabra*, a sea cucumber present in the Indian Ocean and abundantly consumed on the Asian food market. Using mass spectrometry, we first qualitatively and quantitatively assessed the saponin content within the viscera of *H. scabra*. We detected 26 sulfated saponins presenting 5 different elemental compositions. Microwave activation under alkaline conditions in aqueous solutions was developed and optimized to quantitatively and specifically induce the desulfation of the natural saponins, by a specific loss of H_2_SO_4_. By comparing the hemolytic activities of the natural and desulfated extracts, we clearly identified the sulfate function as highly responsible for the saponin toxicity.

## 1. Introduction

Molecules of natural origins have been the focus of scientific interest for years, not only because of their great structural diversity and complexity, but, above all, thanks to their biological properties which, if they are understood and mastered, can be of major industrial interest [1,2]. Among all the different classes of biomolecules, specific (secondary) metabolites, such as flavonoids, alkaloids and saponins, are of major interest for scientific research because of their diverse and specific interactions with diverse living organisms [3,4]. Saponins are specific metabolites that can fulfill defensive roles due to their toxicity, play a role in inter- and intra-species communications [5,6], and even intervene in reproduction processes [7,8]. These molecules are mainly present in the plant kingdom, as shown in a study conducted by Kassem et al. that reveals that 75% of the well-known 1700 varieties of Asian plants and vegetables contain saponins [9]. Saponins were also discovered in diverse marine invertebrates, such as sponges and echinoderms, i.e., sea stars [10] and sea cucumbers [11]. Although the saponin family of molecules is characterized by a great structural diversity, all saponins present a typical molecular pattern consisting in the condensation of one apolar part, named the aglycone, and of a steroidic or triterpenoidic nature, and at least one polar part, the glycan, composed of a linear or branched saccharide chain [12]. Macrocyclic saponins are also described in some sea stars, such as sepositoside A found in *Echinaster sepositus* [13]. Additional chemical groups, such as free carboxylic acid functions (-COOH) [14], esterified acetic, tiglic or angelic acids [15,16], sulfate groups (-OSO_3_H), and many others [10,17,18], are also often encountered on the aglycone and/or the glycan, and may be associated to specific functions to the saponins. The presence or absence of these functions has a strong impact on the amphiphilic character of saponins and, therefore, on their biological properties (antifungal, antibacterial, antitumor, hemolytic, etc.) [19,20,21,22]. Several studies proposed that the saponin’s biological properties are strongly related to their membranolytic activity [23,24,25,26,27]. Saponins indeed strongly interact with membrane sterols, leading to the formation of intra-membrane saponin-sterol complexes [23]. As the membrane concentration of these complexes increases, aggregation phenomena occur on the way to the pore formation, ultimately inducing membrane permeabilization and cell death. The saponin–biological membrane interaction is directly dependent on the structure of the saponins [25,26], as also theoretically demonstrated using computational chemistry [28,29]. Establishing the Structure–Activity Relationship (SAR) of saponins represents a challenging task due to their high structural diversity and the variety of biological activities they are associated with in their ecosystems [23]. An elegant strategy to address such a puzzling task is to specifically modify targeted chemical functions and to evaluate the influence of the performed modifications using a standard biological system, such as red blood cells to evaluate hemolytic activities [22]. As for an example, we recently modulated the membranolytic activity of *Chenopodium quinoa* saponins by fast microwave hydrolysis, transforming the native bidesmosidic saponins into their monodesmosidic analogues [30]. The induced structural modification drastically increases the saponin Hemolytic Activity (HA), which not only makes it possible to obtain saponins of modulated activity, but also to build the SAR of this important family of molecules [30]. Other studies have been conducted to better understand the SAR of saponins, including the esterification of tea saponins [31], the action of various enzymes on *Centella asiatica* saponins [32], or the derivatization of hederagenin [33].

The present study focuses on saponins found in the viscera of the sea cucumber, *Holothuria scabra* Jaeger (1833), a species that is mainly found along the coasts of the Indian Ocean and the western coasts of the Pacific Ocean [34]. *H. scabra* has been extensively harvested over recent centuries by the local populations, and traded within the Asian market as a delicacy in the food industry, and as a stimulant and aphrodisiac in traditional medicine. *H. scabra* has been declared "Endangered" by the IUCN (International Union for Conservation of Nature) [34,35]. Based on a strong partnership between the University of Mons (Belgium) and the University of Toliara (Madagascar), an eco-responsible artificial aquaculture has been developed and helps regulate the market to meet commercial needs [36]. The animal preparation for marketing includes the elimination of the non-valuable viscera to prepare the so-called tradeable trepang. We suggest that this evisceration represents a significant loss of potentially valuable chemicals, including saponins [37]. We recently demonstrated that trepang preparation does not significantly alter the saponin contents, compared to that of fresh animals. However, to the best of our knowledge, the saponin content of the viscera of *H. scabra* is, to date, not reported [37,38,39,40].

**Figure 1:**
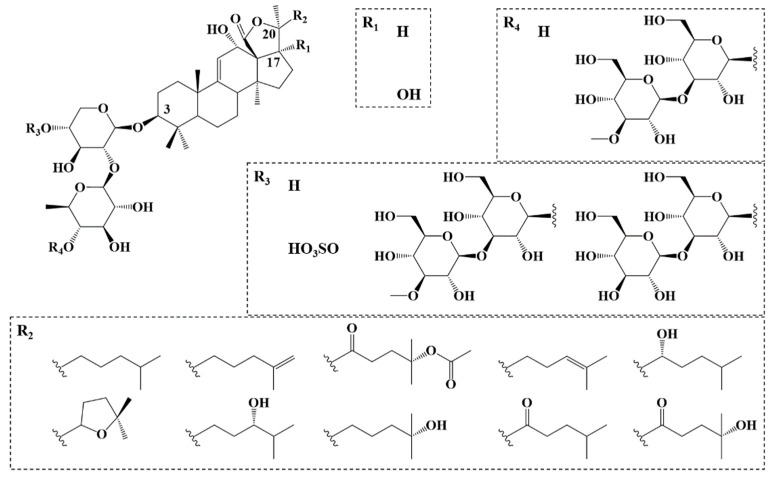
General structure of the *Holothuria scabra* saponins [17] containing a holostanol aglycone with different side groups, including a C3-anchored glycan presenting a sulfate group on the first monosaccharidic unit.

The monodesmosidic triterpene saponins of *H. scabra* consist of a holostanol-based aglycone and a C3-anchored glycan that is either linear or branched, and may contain up to 6 monosaccharide units (see Figure 1) [17,37,38,39,40,41]. Honey-Escandón et al. reported, in their review, the presence of sulfated and non-sulfated saponins in various organs of sea cucumbers, such as the body wall [17]. When present, the sulfate function is invariably anchored on the first saccharide unit of the glycan [17]. The defensive role played by the *H. scabra* saponins (also described as holothurin, desholothurin or scabraside) is believed to be really important for the animal’s survival due to the absence of Cuvierian tubules, that usually represent the main mechanism for the defense/escape of some other species of holothuriid sea cucumbers [42]. Furthermore, the presence of the sulfate group is believed to represent a strong adjuvant to the toxicity of *H. scabra* saponins by increasing their water solubility and also, hypothetically affecting their cytotoxic properties [43,44].

Selectively removing the sulfate group of the *H. scabra* saponins could contribute to validate the hypothetical role of the sulfate group present on these defensive chemicals. Bedini et al., when reviewing the different chemical methods to chemically modify polysaccharides, reported that the efficient desulfation of saccharides may be induced by heating to 80–100 °C in 0.1 M of NaOH aqueous solutions [45].

**Figure 2:**
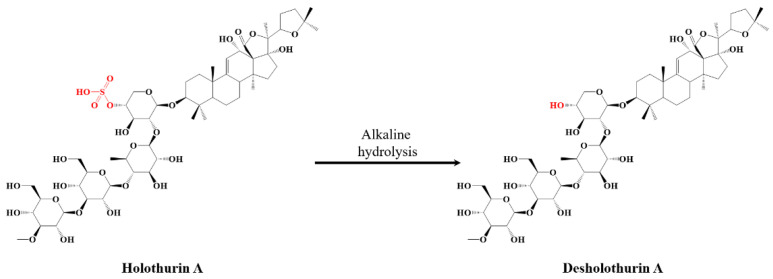
Hypothetical alkaline hydrolysis of Holothurin A leading to Desholothurin A by specific removal of the sulfate group.

In the present study, we intend to investigate whether the saponins present in the viscera of *H. scabra* may be selectively and quantitatively desulfated under microwave conditions, that were demonstrated to afford a fast and homogeneous heating, reaching high selectivity by avoiding side reactions [46]. We will further test the impact of the desulfation on the cytotoxicity of the saponins against mammal red blood cells by comparing the HA of the unmodified and modified saponins. To reach these objectives, we will (i) extract, purify and characterize the saponins from *H. scabra* viscera [17]; (ii) specifically modify (see Figure 2) these saponins by fast microwave activation in aqueous solutions; and (iii) evaluate the HA of the saponins contained in both the natural and hydrolyzed extracts to contribute to the establishment of the SAR of the saponin molecules. State-of-the-art Mass Spectrometry (MS) methods, including Matrix-assisted Laser Desorption/Ionization experiments (MALDI) [47], Liquid Chromatography separation (LC-MS) [48], accurate mass measurements (HRMS—High Resolution MS) [49], and Collision-induced Dissociation experiments (CID or MSMS analysis) [50], will be used to afford the most accurate description of the saponin molecules present in the native and modified extracts. MS is now largely recognized as the standard method for saponin analysis [51].

## 2. Results and Discussion

### 2.1. Saponin Identification and Quantification in the Natural Extract

Saponin characterization requires the combination of the analytical data obtained by MALDI-(HR)MS and LC-MS(MS) [30]. The major *H. scabra* saponins, extracted from the body wall or the internal organs, have already been characterized by Honey-Escandón et al. [17], Caulier et al. [37], Thanh et al. [38], Dang et al. [39], Han et al. [40], and Puspitasari et al. [41] (Figure 1). These studies will help us to characterize most of the saponins present in the viscera extract, obtained as described in the experimental section. Briefly, fresh viscera are dried and ground immediately after collection. The saponins present in the viscera powder are extracted by methanol and further purified using successive liquid/liquid extractions with solvents of increasing polarity, following the standard procedure developed in our laboratory [52]. The saponins are further purified using Flash Chromatography (FC) to generate the saponin extracts that are processed through mass spectrometry analysis, microwave experiments and hemolysis assays. The global yield of extraction/purification is 8.15 mg per 30 g of dried viscera, i.e., ~0.3 mg·g^−1^.

As far as the MS analyses are concerned, the negative ionization mode a priori appears suitable for the detection of negatively charged sulfated saponins. Nevertheless, since the targeted desulfation process will produce saponin molecules deprived of their sulfate group, for consistency reasons, we selected the positive ionization mode for the direct MS data analysis of both the natural and modified saponins. This mode will also allow the detection of putative non-sulfated saponins in the natural extracts.

**Figure 3:**
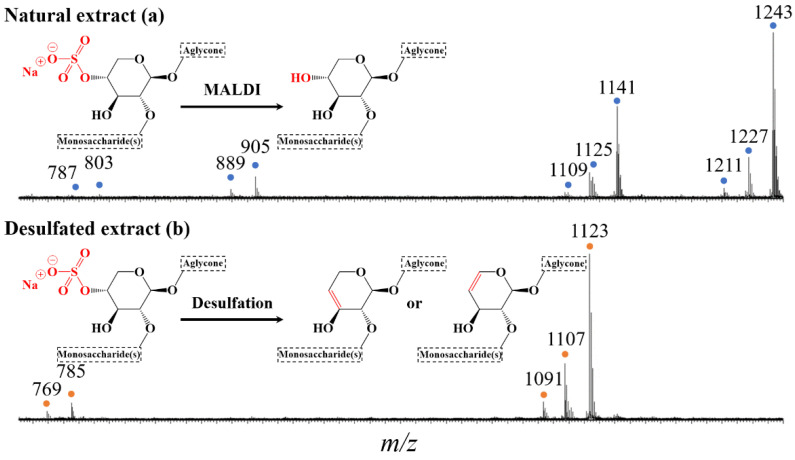
Mass spectrometry analysis of two different saponin extracts: (**a**) the purified saponin extract with detection of sulfated 4-sugar saponin ions, desulfated 4-sugar saponin ions, sulfated 2-sugar saponin ions and desulfated 2-sugar saponin ions; and (**b**) the microwave-assisted alkaline desulfation (pH 14—100 °C—5 min) reaction products with 4-sugar desulfated saponin ions and 2-sugar desulfated saponin ions.

In the MALDI-MS(+) mass spectrum, recorded for the purified extract (after FC), four groups of signals are clearly observed, as featured in Figure 3a. These signals are consistent with the presence of sodium-cationized saponins [M+Na]^+^, based on the literature results presented here above [17,37]. This is further confirmed based on exact mass measurements (MALDI-HRMS(+)) that establish that the detected ions possess the elemental compositions of sulfated 4-sugar saponins (*m*/*z* range 1200–1250), non-sulfated or desulfated 4-sugar saponins (*m*/*z* range 1100–1150), sulfated 2-sugar saponins (*m*/*z* range 875–925), and non-sulfated or desulfated 2-sugar saponins (*m*/*z* range 775–825), generating a total of ten different elemental compositions (see Table 1). Note here that upon MALDI(+) ionization, sulfated saponins appear as [M−H+2Na]^+^ ions, i.e., sodium ion adduct on the sodium sulfate salt (R-OSO_3_Na) of the saponins (in this manuscript, for sulfated saponins, M will systematically represent the acidic saponins as R-OSO_3_H). On the other hand, non-sulfated and desulfated saponins are detected as [M+Na]^+^ ions. The desulfation process detected upon MALDI, corresponds to a SO_3_ loss generating an alcohol function, replacing the sulfate group (see Figure 3a inset). The MALDI experiment alone may not discriminate between non-sulfated and desulfated saponins that, respectively, correspond to saponins that are non-sulfated in the natural extract or desulfated during the MALDI process.

LC-MS(MS) and LC-MS experiments were then conducted to (i) establish that the detected ions are saponin ions, (ii) discriminate between non-sulfated and desulfated saponins, (iii) identify possible (co-eluting) isomers and, (iv) determine the glycan sequence and the nature of the aglycone using CID experiments. During the optimization steps of the LC-MS conditions (solvents: water, HCOOH, CH_3_CN), we observed that protonated saponins [M+H]^+^ are too fragile, and systematically undergo a H_2_O loss, even if the source conditions (including source temperatures and stepwave voltages) of the Waters Synapt G2-S*i* mass spectrometer were carefully tuned. We then slightly modified the composition of the water mobile phase with NaCl (1% *w*/*v*), and [M−H+2Na]^+^ saponin ions then became the dominant species detected upon Electrospray Ionization (ESI), making straightforward the comparison between the MALDI and LC data.

LC-MS(+) analysis demonstrated that the natural saponin extract exclusively contains sulfated saponins with 5 elemental compositions noted A to E, as presented in Table 1 and Figure S1, that gather the MS data generated for the [M−H+2Na]^+^ ions, including (acidic) saponin elemental compositions, HRMS data (MALDI(+)), and LC-MS retention times (RT). The LC-MS experiments also revealed the presence of numerous isomers for each elemental composition, with at least 8 isomers for the A composition, 5 for B, 2 for C, 5 for D and 6 for E. Globally, 26 different sulfated saponins were detected in the natural extract. By integrating the Extracted Ion Current (EIC) chromatograms (Figure S1) for each [M−H+2Na]^+^ ion, the molar ratios of the different isomers can be determined, and are also presented in Table 1. Interestingly, for each elemental composition, one isomer is systematically, significantly more abundant than the others. For instance, for composition A, the saponin eluting after 8.01 min monopolizes around 97% of the molar fraction for the C_54_H_86_O_7_S elemental composition. The most abundant congeners for each elemental composition are highlighted in bold in Table 1 and are arbitrarily named A, B (B_1_ and B_2_), C, D and E (E_1_ and E_2_) in the next sections of the investigation. First of all, the corresponding [M−H+2Na]^+^ ions are submitted to CID experiments (LC-MSMS). The LC-MSMS spectra of the 7 major saponin ions (A–E), allowing the determination of the glycan and the aglycone compositions, are gathered in the Supplementary Information (SI—Figures S2–S9), together with the proposed structures.

**Figure 4:**
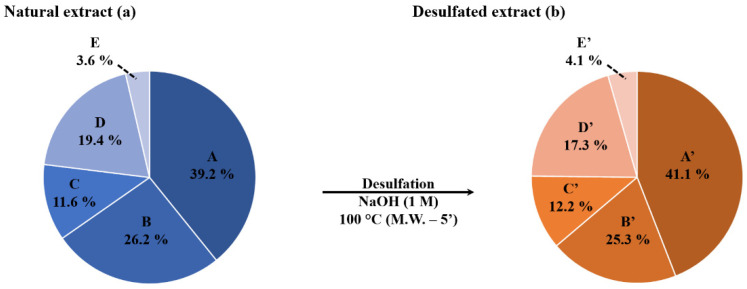
Mass spectrometry qualitative and quantitative analysis of the (**a**) natural and (**b**) desulfated saponin extracts: the saponin relative proportions (%) correspond to the molar proportions in the natural and modified extracts.

Using Hederacoside C as an internal standard, the saponin composition of the saponin extract was determined and expressed in %-weight. The data presented in Table 1 reveal that the saponin molecules presenting the elemental composition A, B, C, D and E, respectively constitute 37.45%, 24.65%, 10.82%, 13.41% and 2.46% in weight of the dried extract, globally amounting to a saponin weight-% of 88.8% within the extract, say 88.8 mg of saponins per 100 mg of dried extract. In Table 1, we also converted the saponin %-weight in the extract into the saponin mass fraction (mg·g^−1^) in the dried viscera, considering that we previously determined that the global yield of extraction/purification is 0.3 mg of extract per g of dried viscera. We then estimated that saponins with elemental compositions A, B, C, D and E, are respectively present up to 0.1, 0.07, 0.03, 0.04 and 0.01 mg per g of dried viscera (Table 1). We recently introduced a representation using sector diagrams, presenting different areas corresponding to the different identified elemental compositions, whose surface proportions represent the molar proportion of each saponin composition in the saponin mixture (Figure 4a). The determination of these molar proportions (Table 1) is based on the integration of the peak surfaces in the corresponding (EIC) LC-MS chromatograms (Figure S1—see experimental section).

### 2.2. Selective Microwave-Assisted Desulfation of Saponins

As exemplified in Figure 2, the main objective of our study is to specifically induce the desulfation of the natural saponins without decomposing the glycan–aglycone skeleton. As recently exploited to successfully hydrolyze the bidesmosidic quinoa saponins [30], we targeted the alkaline desulfation of saponins under microwave conditions, that are often demonstrated to shorten the reaction times due to a faster and more homogeneous heating, while avoiding undesired side reactions [46]. When optimizing the microwave-assisted desulfation of saponins, several parameters must be considered. Typically, the nature of the solvent, the analyte concentration, the pH, the temperature, and the reaction time. We systematically used a concentration of 1 mg·mL^−1^ of extract in aqueous solutions, buffered at a pH between 7 and 14 (see experimental section). We avoided acidic pHs that are known to induce hydrolysis of the glycosidic linkages [53]. The tested temperature range expanded between 80 °C and 110 °C, and the reaction time was modified between 1 and 10 min. All the data are presented in SI (Figures S9–S11), and we observed that quantitative desulfation is reached when submitting the 1 mg·mL^−1^ aqueous solution of the saponin extract at pH 14 to microwave activation at 100 °C for 5 min. Indeed, as shown in the MALDI mass spectrum presented in Figure 3b, all the signals assigned to sulfated/MALDI-desulfated saponins in the MALDI mass spectrum of the natural saponin extract (Figure 3a) are no longer detected after the microwave alkaline treatment, demonstrating that the natural saponins have been chemically modified. The 2-sugar and 4-sugar saponin ions are now detected at *m*/*z* 769 (E’), *m*/*z* 785 (D’), *m*/*z* 1091 (C’), *m*/*z* 1107 (B’) and *m*/*z* 1123 (A’). Upon MALDI-HRMS measurements, the elemental compositions of these ions are readily assigned as a Na^+^ adduct on natural saponins (R-OSO_3_H), formally deprived from an H_2_SO_4_ molecule, see Table 2. It is interesting to compare the MALDI spectra recorded for the untreated and treated saponin extracts, where the MALDI-desulfated saponin ions (SO_3_ formal loss from R-OSO_3_H) and the MW-desulfated saponin ions (H_2_SO_4_ formal loss from R-OSO_3_H) do not possess the same mass-to-charge ratios, pointing to the presence of different elemental compositions.

Using LC-MS analysis, we confirmed that the microwave-assisted desulfation of the saponins is quantitative for all the different saponin congeners present in the natural extract, since the 5 saponin elemental compositions (A–E in Table 1) are now detected as their desulfated counterparts, identified as A’ to E’ in Table 2, with molar fractions that are mostly unaffected by the microwave treatment, see also Figure 4 for the graphical comparison. Moreover, note that, for each elemental composition, different isomers are detected with molar proportions that are marginally affected by the microwave treatment, see Table 1 and Table 2.

**Figure 5:**
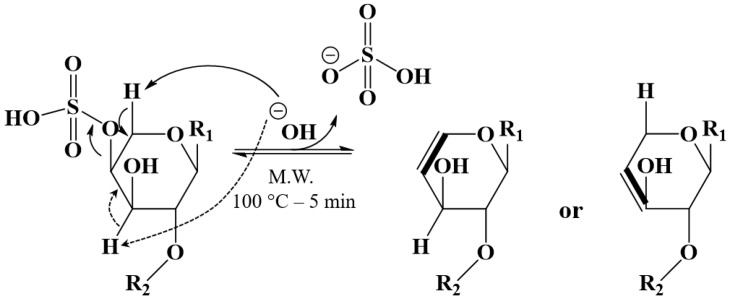
Microwave-assisted alkaline H_2_SO_4_ loss from natural saponins: the C=C bond formation created by the desulfation process involves a vicinal hydrogen atom in the frame of an elimination reaction whose regioselectivity is still ill-defined.

We propose that the formal H_2_SO_4_ loss from the natural saponins corresponds to an elimination reaction involving a vicinal hydrogen atom and the creation of a C=C double bond, as sketched in Figure 5. For the time being, the regioselectivity of the elimination reaction is ill-defined, since two possibilities may be considered. Nevertheless, the quantitative and specific desulfation reaction of the natural saponins is definitively confirmed and we have, thus, now at our disposal two different extracts, treated and untreated, whose compositions are qualitatively and quantitatively defined based on all our MS experiments.

### 2.3. Hemolytic Activity (HA) Modulation

The toxicity of the natural and desulfated saponins are compared based on the determination of the HA of the saponin extracts [27]. Numerous research groups used this fast assay to determine the membranolytic propension of extracted saponins for SAR purposes [22,54,55,56,57]. We recently used this protocol to demonstrate that non-natural monodesmosidic saponins generated by microwave-assisted hydrolysis of the bidesmosidic saponins extracted from the quinoa husk, are significantly more membranolytic than the natural molecules [30]. The HA is measured by submitting a red blood cell suspension to increasing concentrations of the active molecules, and monitoring the membrane lysis by measuring the release of free hemoglobin in the extracellular solution, using a UV-vis spectroscopy at 540 nm [58]. As described in the experimental section, the HA of a given extract is then compared to a referent solution (500 µg·mL^−1^ of Horse Chestnut (HC) saponins) as detailed in the experimental section and expressed in % of the HC solution activity [59].

**Figure 6:**
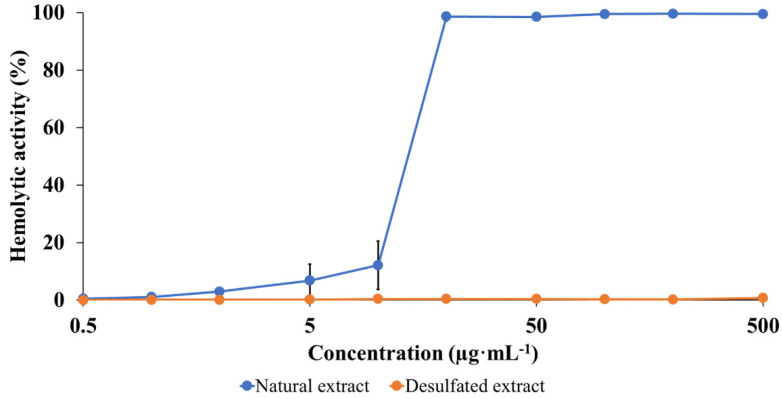
Evaluation of the cytotoxicity of both saponin extracts: comparison between the hemolytic activity of the natural (sulfated) saponins and of the microwave-desulfated saponins. The experiment was performed in triplicates using a 2% suspension of erythrocytes from bovine blood. The HAs are expressed in % of the HE of a reference extract constituted by 500 µg·mL^−1^ of HC saponins (see experimental).

The data presented in Figure 6 immediately confirm that the sulfate group plays a crucial role in the membranolytic activity of *H. scabra* saponins. Indeed, we note that the desulfated saponins remain inactive, even at a concentration of 500 µg·mL^−1^ that corresponds to the solubility limit of the saponins in the red blood cell suspension. On the contrary, the natural saponins start to be active against red blood cells already at 1 µg·mL^−1^, and the 540 nm absorbance steadily increases with increasing saponin concentration.

## 3. Materials and Methods

### 3.1. Chemicals

All solvents, i.e., technical grade methanol, hexane, chloroform, dichloromethane, isobutanol, HPLC grade water, and HPLC grade acetonitrile were provided by CHEM-LAB NV (Somme-Leuze, Belgium). Hederacoside C, 2,5-dihydroxybenzoic acid (DHB) and N,N-dimethylaniline (DMA) were purchased from Sigma-Aldrich (Diegem, Belgium). Sodium citrate, sodium chloride, disodium phosphate dihydrate, potassium chloride, monopotassium phosphate, and borax were provided by VWR Chemicals (Leuven, Belgium). 

### 3.2. Extraction & Purification

The viscera (comprising the intestinal tube, the respiratory trees, and the male and female gonads) of *H. scabra* were recovered following the evisceration of sea cucumbers collected in spring 2018 at the Indian Ocean Trepang (IOT) sea cucumbers farm in Madagascar. They were then placed in direct sunlight for seven days till complete dryness. The dried samples were brought back to UMONS, and kept in the fridge at a temperature of 4 °C. Thereafter, the viscera (30 g) were placed overnight in an oven at 50 °C in order to complete the drying process. The extraction of the saponins began with the grinding of the viscera using an IKA A 11 grinder. The ground material was collected and placed under stirring overnight in methanol. After that, the solution was centrifuged at 4500× *g* rpm for five minutes (Sigma 2-16P). The supernatant was then collected and diluted with water to reach a methanol/water volume ratio of 70/30. Three liquid/liquid extractions (*v*/*v*) were successively carried out with *n*-hexane (C_6_H_14_), chloroform (CHCl_3_), and dichloromethane (CH_2_Cl_2_) to remove apolar molecules. The third aqueous phase was recovered in a flask and evaporated under vacuum using the rotary evaporator IKA RV 10 at 80 rpm in a water bath at 50 °C. The collected residue was brought to a volume of 25 mL using Milli-Q water. A fourth liquid/liquid decantation (*v*/*v*) was carried out with HPLC isobutanol (C_4_H_10_O) overnight, in order to conserve the saponins in the organic phase and to purify the extract from the salts migrating to the aqueous phase. The organic phase was then evaporated under vacuum and the residue was dissolved in 10 mL of Milli-Q water. The solution was then passed to flash chromatography (Biotage SP1 Flash Chromatograph—Biotage Sweden, Uppsala, Sweden) on a non-polar column (Büchi Cartouche FlashPure ID C18-WP Flash—BUCHI Labortechnik GmbH, Hendrick-Ido-Ambracht, The Netherlands). The solvent was composed of a water and technical acetonitrile (CH_3_CN) mixture. The sample injected into the chromatography column was diluted in the mobile phase and fractionated to a defined volume of 15 mL. The fractions of interest were then pooled and evaporated. The residue was brought to a volume of 20 mL with Milli-Q water and the solution was brought to pH 7 using a 0.5 M solution of potassium hydroxide (KOH). The solution underwent a fifth and final liquid/liquid (overnight) decantation (*v*/*v*) with HPLC isobutanol, to separate the saponins from the residual salts. The organic phase was evaporated to dryness to recover the mixture of purified saponins in the form of a slightly yellowish powder.

### 3.3. Microwave-Assisted Alkaline Desulfation

The hydrolysis protocol was adapted from Bedini et al. [45]. *H. scabra* natural extract (3 mg) is solubilized in 3 mL of different buffer solution covering a pH range from 7 to 14. For pH 7: 50 mL of KH_2_PO_4_ 0.1 mol·L^−1^ is added to 29.1 mL of NaOH 0.1 mol·L^−1^ and brought to 100 mL with Milli-Q water. For pH 8: 50 mL of KH_2_PO_4_ 0.1 mol.L^−1^ is added to 46.7 mL of NaOH 0.1 mol·L^−1^ and brought to 100 mL with Milli-Q water. For pH 9: 50 mL of borax 0.025 mol.L^−1^ is added to 4.6 mL of HCl 0.1 mol·L^−1^ and brought to 100 mL with Milli-Q water. For pH 10: 50 mL of borax 0.025 mol.L^−1^ is added to 18.3 mL of NaOH 0.1 mol.L^−1^ and brought to 100 mL with Milli-Q water. For pH 11: 50 mL of Na_2_HPO_4_ 0.05 mol·L^−1^ is added to 4.1 mL of NaOH 0.1 mol·L^−1^ and brought to 100 mL with Milli-Q water. For pH 12: 50 mL of Na_2_HPO_4_ 0.05 mol·L^−1^ is added to 26.9 mL NaOH 0.1 mol·L^−1^ and brought to 100 mL with Milli-Q water. For pH 13: 0.4 g of NaOH is dissolved in 100 mL Milli-Q water to reach mol·L^−1^. For pH 14: 4 g of NaOH is dissolved in 100 mL Milli-Q water to reach mol·L^−1^.

Samples are heated at different temperatures for different reaction times using a microwave device (Biotage, Initiator Classic, Biotage Sweden, Uppsala, Sweden). Once cooled to room temperature, the pH is brought to 7 with hydrochloric acid (0.1 mol·L^−1^) and an extraction with isobutanol is performed (*v*/*v*). The organic phase is then washed twice with water to remove residual salts and evaporated under vacuum to recover the product in powder form (85% yield).

### 3.4. Mass Spectrometry

Mass spectrometry is used (i) to select the FC fractions containing saponins and (ii) to determine the composition of the extracts qualitatively and quantitatively.

These analyses were carried out by Matrix-assisted Laser Desorption/Ionization (MALDI) and were performed on a Waters QToF Premier mass spectrometer (Waters, Manchester, UK) in positive ion mode, MALDI-MS(+). The matrix consisted of a mixture of dihydroxybenzoic acid (DHB, 25 mg) and N,N-dimethylaniline (DMA, 6 µL, 99.9%) in 250 µL of acetonitrile/water (*v*/*v*). A droplet of matrix solution (1 µL) was placed on a stainless-steel plate and dried in the open air before depositing a droplet of solution to be analyzed (1 µL). The plate was then introduced into the MALDI-ToF spectrometer once the co-crystal has been formed. The MALDI source was composed of an Nd-YAG laser with a maximum energy of 104.1 µJ, transferred to the sample in a 2.2 ns pulse at a frequency of 200 Hz. For spectral recording, the quadrupole in rf-only mode was configured to let the ions pass between *m*/*z* 250 and 2500. All the ions were then sent to the push/puller of the ToF analyzer, where they were mass analyzed with a 1 s integration time. Mass analyses were performed with the ToF analyzer in reflectron mode, providing a resolution close to 10,000. Accurate mass measurements (HRMS) were performed using MALDI-MS(+) with PEG 600–1500 as the external standard (lock mass).

In order to identify the presence of isomers and quantify their relative proportions, liquid chromatography analyses were performed with a Waters Acquity UPLC H-Class (Waters, Manchester, UK), composed of a vacuum degasser, a quaternary pump and an autosampler, coupled to the Waters Synapt G2-S*i* mass spectrometer (Waters, Manchester, UK). This introduction technique allows separation of the compounds according to their affinity with a non-polar column (Acquity UPLC BEH C18; 2.1 × 50 mm; 1.7 µm; Waters, Manchester UK), at 40 °C. For these analyses, 0.1 mg of saponin extract was dissolved in 1 mL of a Milli-Q water/acetonitrile solution (85/15). A volume of 5 µL was injected on the column. The gradient was optimized for the compounds in this study and follows a flow rate of 250 µL.min^−1^ of Milli-Q water, with 0.1% formic acid (HCOOH), and acetonitrile (CH_3_CN). The mobile phase consisted of an elution gradient starting with 85% of eluent A and 15% of eluent B, and reaching 60% of eluent A and 40% eluent B at 6 min, and maintained for 3 min. The ratio was then modified to reach 5% eluent A and 95% eluent B at 11 min, maintained for 1 min and, finally, brought back to 85% eluent A and 15% eluent B at 13 min. This ratio was maintained until the end of the chromatographic run (15 min). The saponin ions were then produced by means of Electrospray Ionization (ESI) in positive ionization mode. The analyses were performed applying the following conditions: capillary voltage 3.1 kV, cone voltage 40 kV, source offset 80 V, source temperature of 120 °C and a desolvation temperature of 300 °C (dry nitrogen flow rate 500 L.h^−1^), for a mass range (quadrupole in rf-only mode) between *m*/*z* 50 and 2000, with an integration time of 1 s. For the LC-MSMS experiments, precursor ions were mass selected by the quadrupole and collided against argon (Ar) in the Trap cell of the Tri-Wave device, and the kinetic energy of the laboratory frame (E_lab_) was selected to afford intense enough product ion signals. The fragment ions were mass-measured with the ToF analyzer.

The relative quantification of natural extract was achieved by adding a known quantity (0.1 mg·mL^−1^) of commercially available Hederacoside C (Sigma-Aldrich—Product n° 97151—M-Clarity^TM^ Program MQ100), a pure saponin from *Hedera helix*, as internal standard in a solution of saponin extract at a given concentration, typically 0.1 mg·mL^−1^. The spiked solution was analyzed using LC-MS (injection of 5 µL), using the experimental conditions described here above. For each saponin molecule, including Hederacoside C, the corresponding LC-MS ion signals—including all the isotopic compositions—were integrated using the integration algorithm, available under MassLynx^TM^ 4.1 Software. The global ion counts were then used to estimate the concentration of each saponin, relatively, to Hederacoside C signal integration. The %-weights in extract (see Table 1) correspond to the mass percentages of saponins with a given elemental composition within the saponin extract. Note that the sum of the %-weight does not reach 100%, allowing one to estimate the saponin content within the extract at 88.8%. The Mass Fractions in viscera (see Table 1) are further calculated by using the global yield of extraction/purification determined at 0.3 mg of extract per g of dried viscera.

### 3.5. Hemolytic Activity Assay

To measure the HA, reflecting the membranolytic activity, bovine blood (stored with 0.11 M sodium citrate) was collected immediately after the death of the animal at the “Abattoirs de Ath” (22 Chemin des Peupliers, 7800 Ath, Belgium) on 3 April 2021. The erythrocytes were then washed using a Phosphate Buffered Saline (PBS) solution. This solution was prepared by dissolving 8 g of sodium chloride (NaCl), 1.45 g of sodium hydrogen phosphate dihydrate (Na_2_HPO_4_·2H_2_O), 0.2 g of potassium chloride (KCl) and 0.2 g potassium dihydrogen phosphate (KH_2_PO_4_) in 800 mL of Milli-Q water. The solution was brought to pH 7.4 and made up to volume (1 L) using Milli-Q water. In a 50 mL Falcon, 10 mL of citrated bovine blood were added to 40 mL of PBS solution. The Falcons were then centrifuged for fifteen minutes at 10,000× *g* and the pellet was preserved. This washing was repeated until a clear and colorless supernatant was obtained. The supernatant was discarded, and a 2% (*v*/*v*) erythrocyte suspension was prepared by diluting 2 mL from the pellets to 98 mL of PBS. At the same time, various solutions containing the extract of saponins at different concentrations were prepared. The latter were placed in the presence of the 2% erythrocyte suspension in triplicate and incubated for one hour at 20 °C, with continuous shaking (500 rpm) before being centrifuged again at 10,000× *g* for ten minutes. The supernatant of each sample was then collected to measure the absorbance of the solution at 540 nm [60]. In our assay, we systematically used a 500 µg·mL^−1^ solution of saponins extracted from *Aesculus hippocastanum* seeds as a reference solution, since the corresponding escins are highly membranolytic [59]. The HA of the tested saponin solutions were then calculated using the following equation:

(1) $$\mathrm{HA}\;\left(\%\right)=\frac{\left({\mathrm{Abs}}_{\mathrm{sample}}-{\mathrm{Abs}}_{\mathrm{blank}}\right)}{\left({\mathrm{Abs}}_{\mathrm{ref}}-{\mathrm{Abs}}_{\mathrm{blank}}\right)}*100$$

where Abs_sample_, Abs_blank_, and Abs_ref_ respectively correspond to the absorbance at 540 nm of the tested erythrocytes/saponins solutions, of the erythrocytes solution and of the erythrocytes/referent saponin solution.

## 4. Conclusions

The saponin content within the *H. scabra* viscera has been qualitatively and quantitatively determined using a combination of mass spectrometry methods including MALDI-(HR)MS and LC-MS(MS) experiments. We detected 26 sulfated saponins presenting 5 different elemental compositions. We also observed that the desulfation of natural saponins, i.e., SO_3_ loss, readily occurs under MALDI conditions, and that protonated sulfated saponins, generated under Electrospray conditions, are quite fragile and quantitatively suffer a water loss. Intact sulfated saponins are only detected as [M−H+2Na]^+^ ions, whose production is forced by adding Na^+^ in the LC mobile phase. Using Hederacoside C as an internal standard for the quantification, we estimated that the saponin content amounts to ~ 90% in weight of the dried purified extract. The hemolytic activity of this extract has been evaluated against red blood cells, and saponin solutions are already active against erythrocytes at concentrations as low as 1 µg·mL^−1^ in the 2% erythrocyte suspension.

We then induced the saponin desulfation by microwave activation and we optimized the reaction (temperature, pH, reaction time) conditions to reach a quantitative and specific desulfation. Using mass spectrometry, we also established that such a desulfation reaction actually corresponds to an H_2_SO_4_ elimination, creating a C=C double bond in the monosaccharide ring. These non-natural saponins were further shown to be no longer hemolytic, since no HA is measured, even at a concentration of 500 µg·mL^−1^, that corresponds to the solubility limit of the saponins in the red blood cell suspension.

In conclusion, the present study demonstrates that saponins extracted from the viscera of *H. scabra* exhibit significant cytotoxicity, directly induced by the presence of the sulfate function. The results also reveal that it is possible to modulate the cytotoxicity of saponins. These modified saponins are currently assayed against different bacteria and fungi since non hemolytic saponins with antifungal and/or bactericide properties will be appealing for biomedical and pharmaceutical applications.

## Figures and Tables

**Table 1:** *Holothuria scabra* viscera extract: data collected by MS-based methods. The compositions and the mass error measurements (Δ) were determined by MALDI-HRMS. The Mass fractions (mg·g^−1^ of *Holothuria scabra* viscera powder) of each saponin composition were determined based on the LC ion signal intensity ratios, with Hederacoside C as an internal standard. Note that the MALDI-desulfated saponins are not considered in the present table that only gathers the natural extract saponins.

Saponin	Composition (M)	*m*/*z* (Δ ppm) [M−H+2Na]^+^	%-Weight in Extract (%)	Mass Fraction in Viscera (mg·g^−1^)	Retention Time (min)	Composition Molar Proportion (%)	Isomer Molar Proportion (%)
A	C_54_H_86_O_27_S	1243.4795 (1.3)	37.45 ± 0.37	0.102	5.97	39.18 ± 1.24	0.57 ± 0.11
					6.24		0.75 ± 0.11
					6.48		0.11 ± 0.02
					6.76		0.20 ± 0.02
					7.20		0.48 ± 0.07
					7.81		0.21 ± 0.04
					**8.01**		**97.54 ± 0.38**
					8.74		0.14 ± 0.02
B	C_54_H_86_O_26_S	1227.4845 (1.5)	24.65 ± 1.04	0.067	6.09	26.14 ± 0.56	0.67 ± 0.11
					6.34		0.78 ± 0.14
					**8.47**		**26.87 ± 0.41**
					11.01		2.77 ± 1.40
					**11.16**		**68.91 ± 0.88**
C	C_54_H_86_O_25_S	1211.4896 (1.2)	10.82 ± 0.84	0.029	11.14	11.64 ± 1.22	3.80 ± 0.11
					**11.26**		**96.20 ± 0.11**
D	C_41_H_64_O_17_S	905.3582 (1.2)	13.41 ± 0.20	0.036	7.19	19.41 ± 1.04	0.92 ± 0.04
					7.56		0.97 ± 0.08
					9.25		0.32 ± 0.03
					9.37		0.37 ± 0.01
					**11.02**		**97.42 ± 0.15**
E	C_41_H_64_O_16_S	889.3632 (3.1)	2.46 ± 0.67	0.007	7.29	3.63 ± 0.32	1.96 ± 0.59
					7.37		1.66 ± 0.45
					7.74		4.26 ± 0.81
					**11.36**		**53.64 ± 2.89**
					12.04		0.85 ± 0.16
					**12.16**		**37.63 ± 2.81**

**Table 2:** Microwave-assisted alkaline desulfation (pH 14—100 °C—5 min) of *Holothuria scabra* viscera saponin extract: the saponin ion’s elemental compositions are determined by MALDI-HRMS. The Composition Molar proportion (%) of each composition and the isomer molar proportions are estimated based on the ion signal ratios, as determined by mass spectrometry experiments (LC-MS).

Saponin	Composition	*m*/*z* (Δ ppm) [M+Na]^+^	Retention Time (min)	Composition Molar Proportion (%)	Isomer Molar Proportion (%)
A’	C_54_H_84_O_23_	1123.5301 (0.7)	7.60 7.90 8.76 8.89 10.26 11.16 **11.50** 11.68	41.08 ± 0.26	0.93 ± 0.06
					0.91 ± 0.04
					0.59 ± 0.09
					0.66 ± 0.09
					1.88 ± 0.06
					6.47 ± 0.22
					**87.66 ± 0.08**
					0.90 ± 0.16
B’	C_54_H_84_O_22_	1107.5376 (1.9)	8.39 8.61 9.13 **11.67** **12.04**	25.34 ± 0.53	2.70 ± 0.12
					2.42 ± 0.11
					7.71 ± 0.16
					59.82 ± 0.83
					27.35 ± 0.80
C’	C_54_H_84_O_23_	1091.5403 (1.8)	**12.11** 12.24	12.18 ± 0.32	**96.37 ± 0.15**
					3.63 ± 0.15
D’	C_41_H_62_O_13_	785.4038 (2.8)	8.67 9.30 11.65 **11.87** 12.01	17.34 ± 0.51	0.43 ± 0.03
					0.79 ± 0.05
					4.64 ± 0.08
					**93.56 ± 0.05**
					0.58 ± 0.01
E’	C_41_H_63_O_12_	769.4139 (0.5)	10.18 10.75 **10.97** 11.62 **12.07** 12.43	4.06 ± 0.26	2.06 ± 0.82
					2.12 ± 0.43
					**36.44 ± 0.69**
					3.36 ± 0.22
					**51.98 ± 0.18**
					4.04 ± 0.43

## Data Availability

The data presented in this study are available on request from the corresponding author.

## Supplementary Materials

The following supporting information can be downloaded at, Figure S1: LC-MS analysis of the natural saponin extract: EIC (Extracted Ion Current Chromatogram) of *m*/*z* 1243, *m*/*z* 1227, *m*/*z* 1211, *m*/*z* 905, and *m*/*z* 889, respectively corresponding to [M−H+2Na]^+^ ions of extracted sulfated saponins from *Holothuria scabra* viscera; Figure S2: LC-MSMS(+) analysis of *Holothuria scabra* viscera saponin extract: CID spectrum (75 eV), recorded for the *m*/*z* 1243 precursor ions [M−H+2Na]^+^ at 8.01 min retention time (A); Figure S3: LC-MSMS(+) analysis of *Holothuria scabra* viscera saponin extract: CID spectrum (75 eV), recorded for the *m*/*z* 1227 precursor ions [M−H+2Na]^+^ at 8.47 min retention time (B_1_); Figure S4: LC-MSMS(+) analysis of *Holothuria scabra* viscera saponin extract: CID spectrum (75 eV), recorded for the *m*/*z* 1227 precursor ions [M−H+2Na]^+^ at 11.16 min retention time (B_2_); Figure S5: LC-MSMS(+) analysis of *Holothuria scabra* viscera saponin extract: CID spectrum (75 eV), recorded for the *m*/*z* 1211 precursor ions [M−H+2Na]^+^ at 11.26 min retention time (C); Figure S6: LC-MSMS(+) analysis of *Holothuria scabra* viscera saponin extract: CID spectrum (60 eV), recorded for the *m*/*z* 905 precursor ions [M−H+2Na]^+^ at 11.02 min retention time (D); Figure S7: LC-MSMS(+) analysis of *Holothuria scabra* viscera saponin extract: CID spectrum (60 eV), recorded for the *m*/*z* 889 precursor ions [M−H+2Na]^+^ at 11.36 min retention time (E_1_); Figure S8: LC-MSMS(+) analysis of *Holothuria scabra* viscera saponin extract: CID spectrum (60 eV), recorded for the *m*/*z* 889 precursor ions [M−H+2Na]^+^ at 12.16 min retention time (E_2_); Figure S9: Microwave-assisted desulfation of *Holothuria scabra* sulfated saponins (150 °C for 10 min): influence of the pH (7 to 14) on the desulfation reactions as estimated by LC-MS(+); Figure S10: Microwave-assisted desulfation of *Holothuria scabra* sulfated saponins (pH 14 for 3 min): influence of the temperature (80 to 110 °C) on the desulfation reactions as estimated by LC-MS(+); Figure S11: Microwave-assisted desulfation of *Holothuria scabra* sulfated saponins (pH 14 at 100 °C): influence of the time (1 s to 10 min) on the desulfation reactions as estimated by LC-MS(+).

## Sample Availability

Samples of the compounds are not available from the authors.
