# Supplementary material for: Microwave-Assisted Desulfation of the Hemolytic Saponins Extracted from Holothuria scabra Viscera
Source: Molecules. 2022 Jan 15;27(2):537. doi: 10.3390/molecules27020537 (PMC8780253; doi:10.3390/molecules27020537)
Supplement: Supplementary file 1 [file molecules-27-00537-s001.zip › molecules-1552190-supplementary.pdf]

Microwave-Assisted Desulfation of the Hemolytic Saponins Extracted from *Holothuria scabra* Viscera

Philippe Savarino [1], Emmanuel Colson [1], Guillaume Caulier [2,3], Igor Eeckhaut [2,3], Patrick Flammang [2] and Pascal Gerbaux [1,*]

[1] Organic Synthesis and Mass Spectrometry Laboratory (S²MOs), University of Mons, 23 Place du Parc, 7000 Mons, Belgium; philippe.savarino@umons.ac.be (P.S.); colson-emmanuel@outlook.be (E.C.)

[2] Biology of Marine Organisms and Biomimetics Unit (BOMB), Research Institute for Biosciences, University of Mons, 23 Place du Parc, 7000 Mons, Belgium; guillaume.caulier@umons.ac.be (G.C.); igor.eeckhaut@umons.ac.be (I.E.); patrick.flammang@umons.ac.be (P.F.)

[3] Belaza Marine Station, Institut Halieutique et des Sciences Marines (IH.SM), University of Toliara, Rue Dr Rabesandratana HD, P.O. Box 141, 601 Toliara, Madagascar

* Correspondence: pascal.gerbaux@umons.ac.be

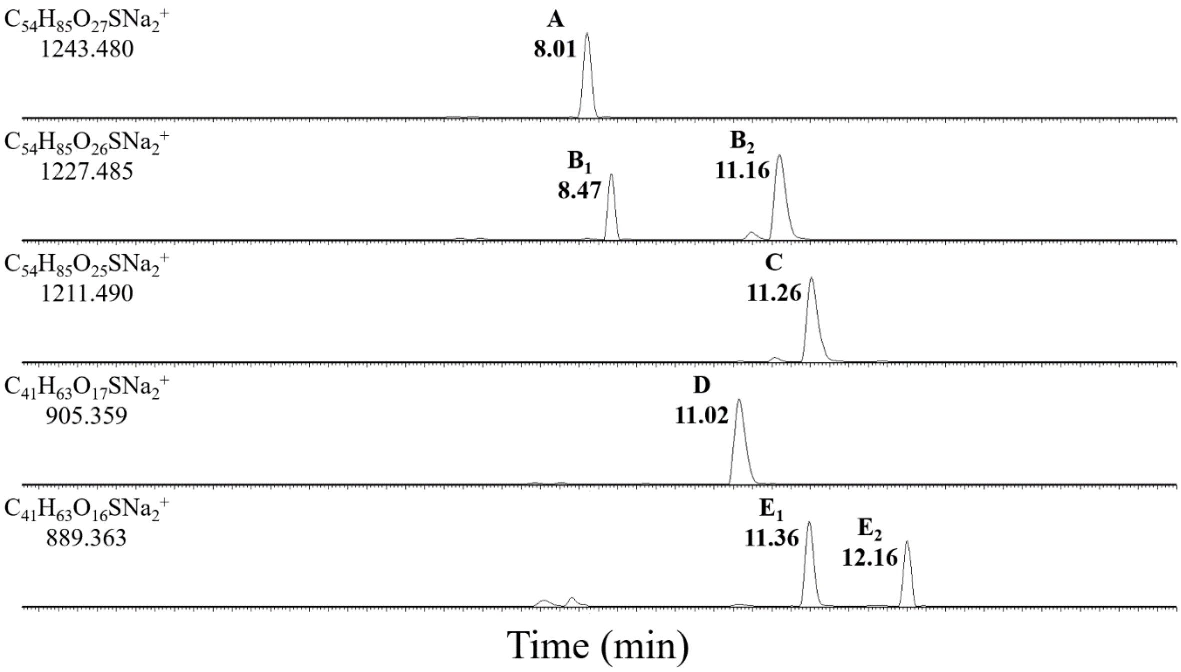

Figure S1: LC-MS analysis of the natural saponin extract : EIC (Extracted Ion Current Chromatogram) of *m/z* 1243, *m/z* 1227, *m/z* 1211, *m/z* 905, and *m/z* 889, respectively corresponding to $[M-H+2Na]^+$ ions of extracted sulfated saponins from *Holothuria scabra* viscera

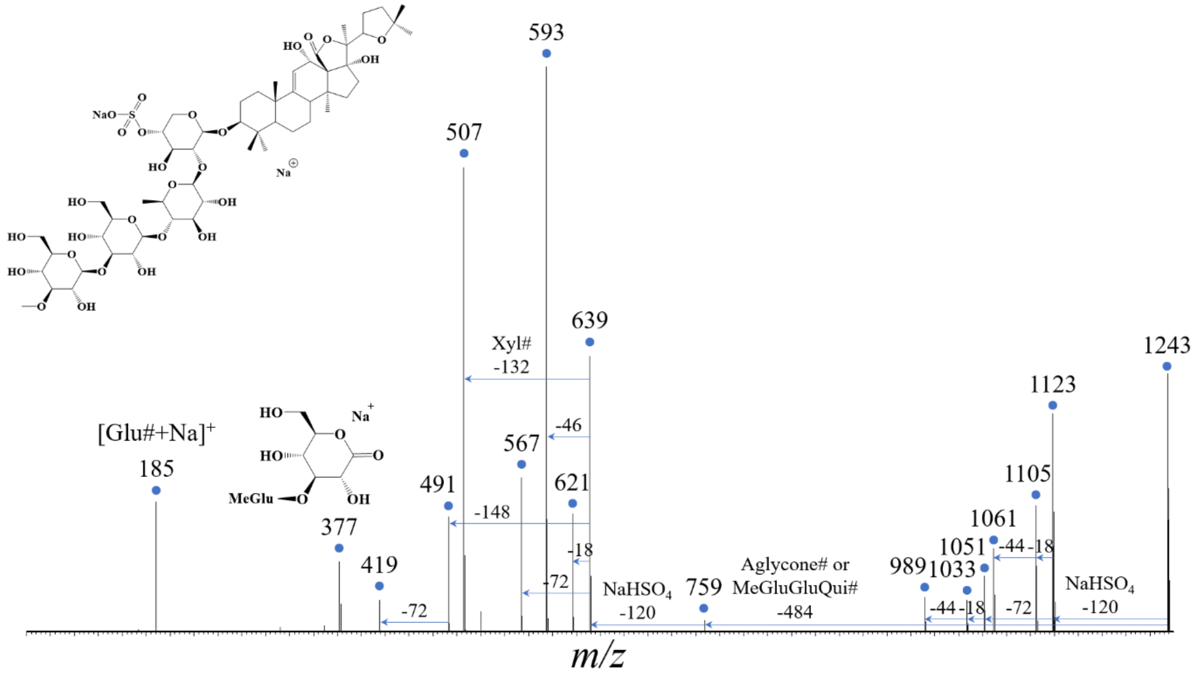

Figure S2: LC-MSMS(+) analysis of *Holothuria scabra* viscera saponin extract: CID spectrum (75 eV) recorded for the *m/z* 1243 precursor ions $[M-H+2Na]^+$ at 8.01 min retention time (A)

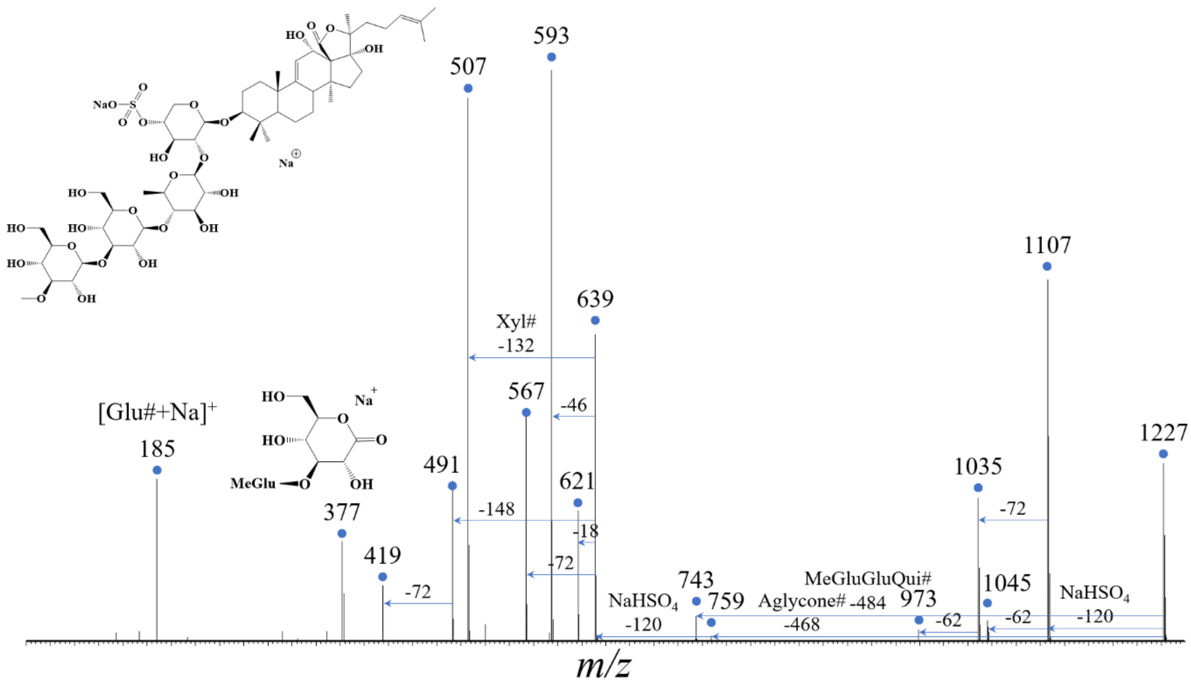

Figure S3: LC-MSMS(+) analysis of *Holothuria scabra* viscera saponin extract: CID spectrum (75 eV) recorded for the *m/z* 1227 precursor ions $[M\text{-}H\text{+}2Na]^+$ at 8.47 min retention time (B1)

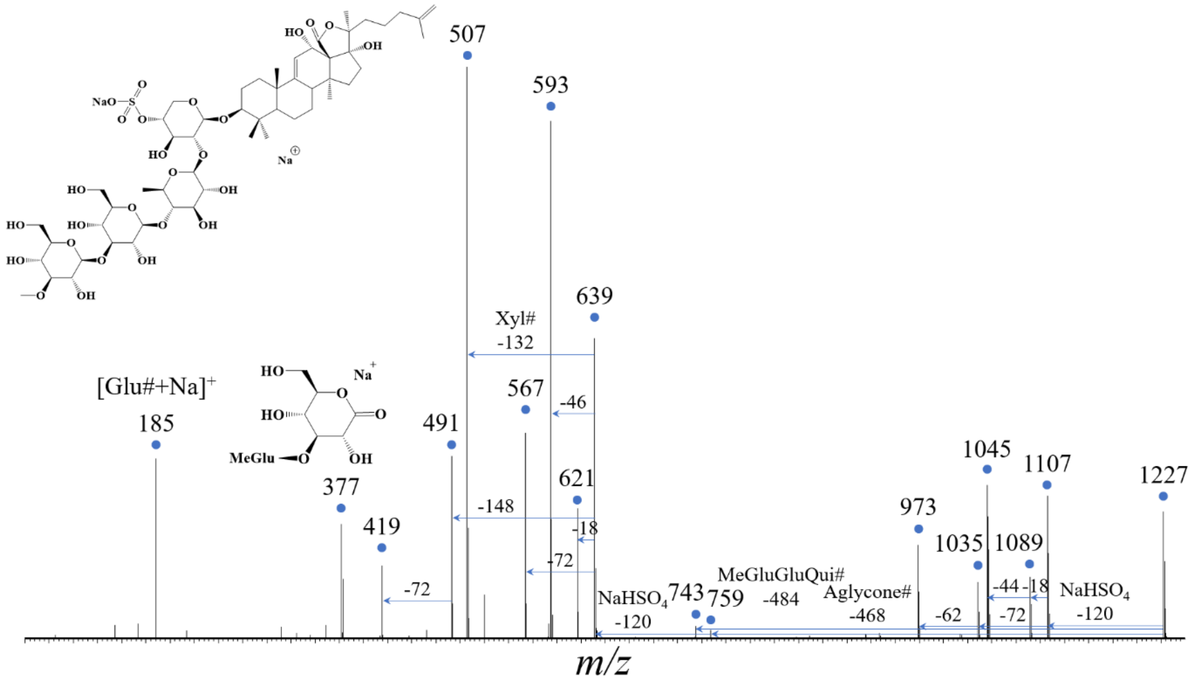

Figure S4: LC-MSMS(+) analysis of *Holothuria scabra* viscera saponin extract: CID spectrum (75 eV) recorded for the *m/z* 1227 precursor ions $[M\text{-}H\text{+}2Na]^+$ at 11.16 min retention time (B2)

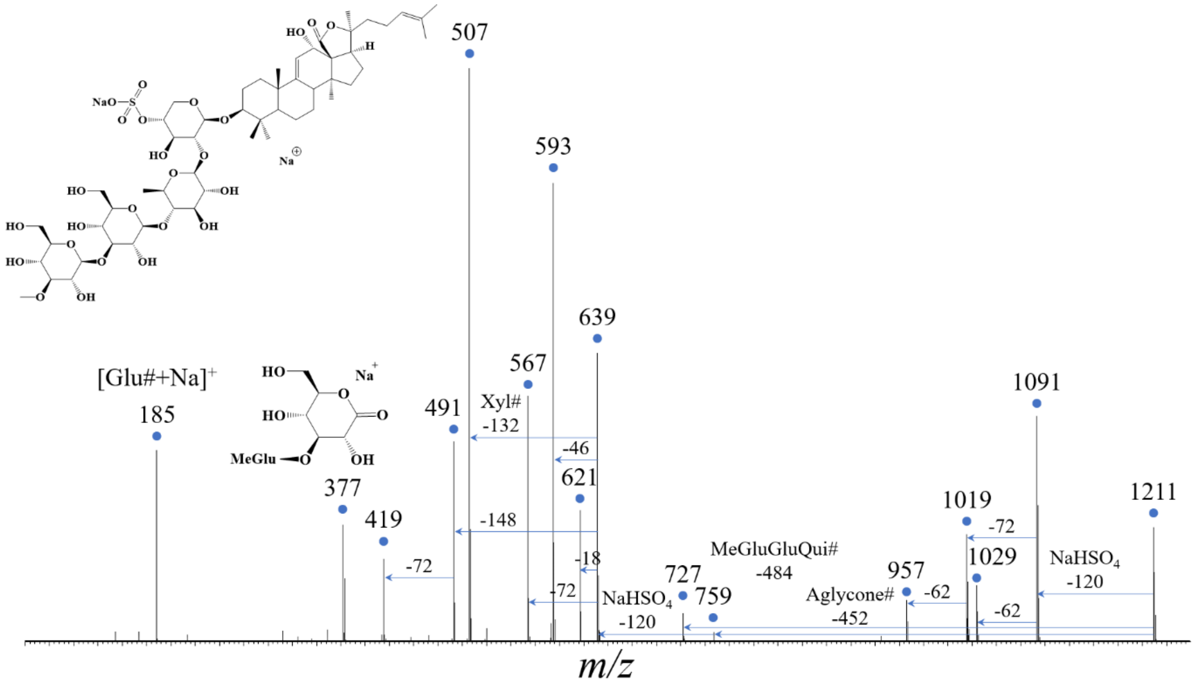

Figure S5: LC-MSMS(+) analysis of *Holothuria scabra* viscera saponin extract: CID spectrum (75 eV) recorded for the *m/z* 1211 precursor ions [M-H+2Na]+ at 11.26 min retention time (C)

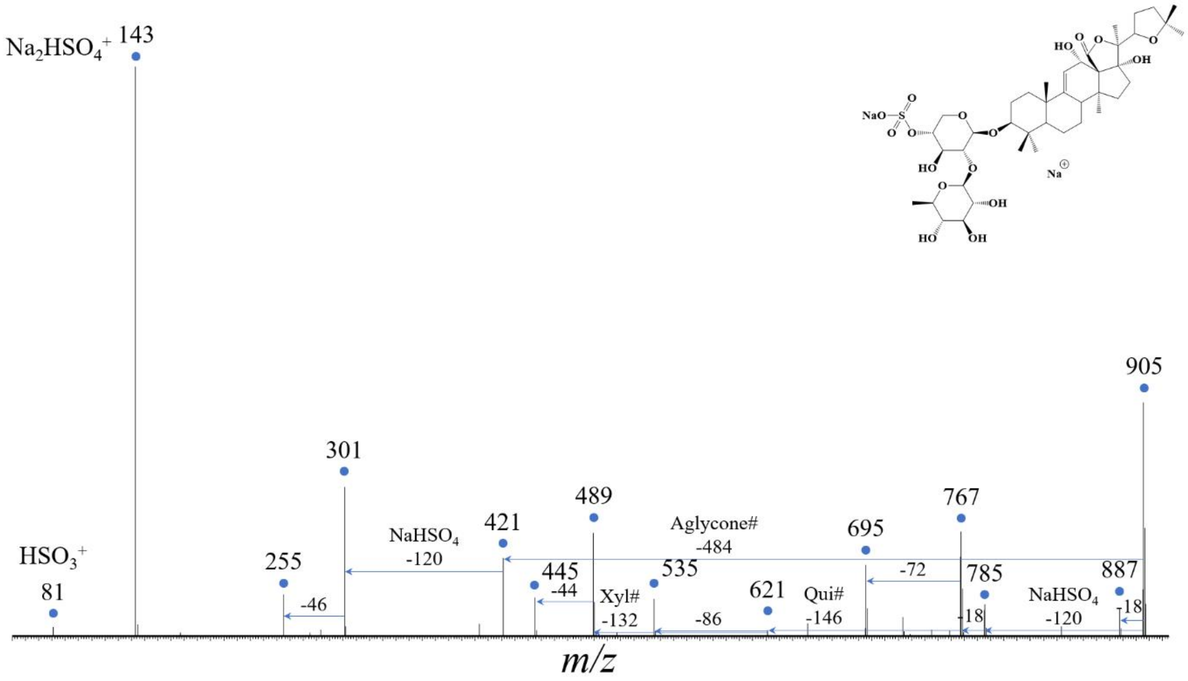

Figure S6: LC-MSMS(+) analysis of *Holothuria scabra* viscera saponin extract: CID spectrum (60 eV) recorded for the *m/z* 905 precursor ions [M-H+2Na]+ at 11.02 min retention time (D)

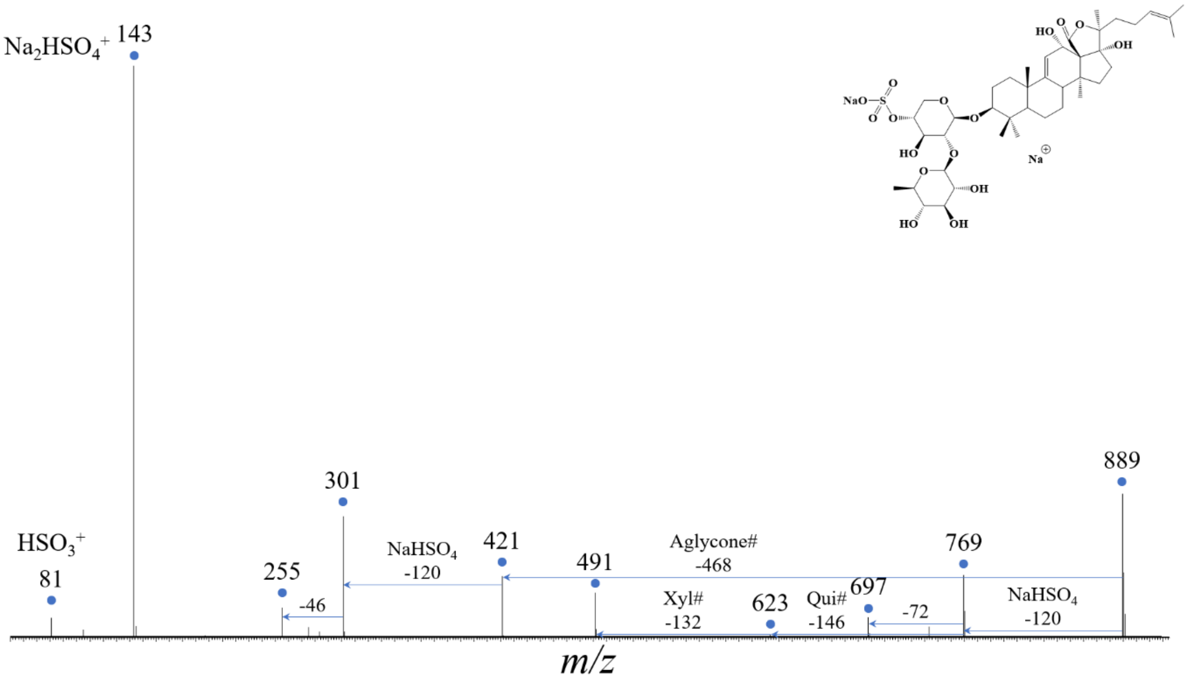

Figure S7: LC-MSMS(+) analysis of *Holothuria scabra* viscera saponin extract: CID spectrum (60 eV) recorded for the *m/z* 889 precursor ions [M-H+2Na]⁺ at 11.36 min retention time (E1)

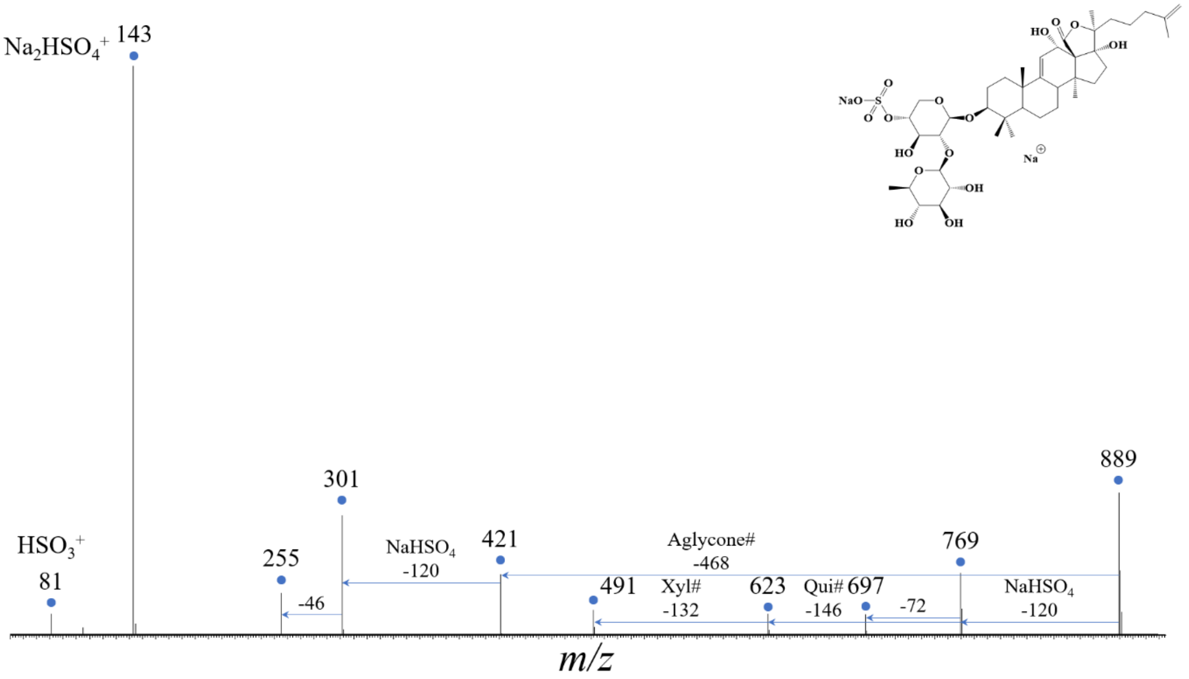

Figure S8: LC-MSMS(+) analysis of *Holothuria scabra* viscera saponin extract: CID spectrum (60 eV) recorded for the *m/z* 889 precursor ions [M-H+2Na]⁺ at 12.16 min retention time (E2)

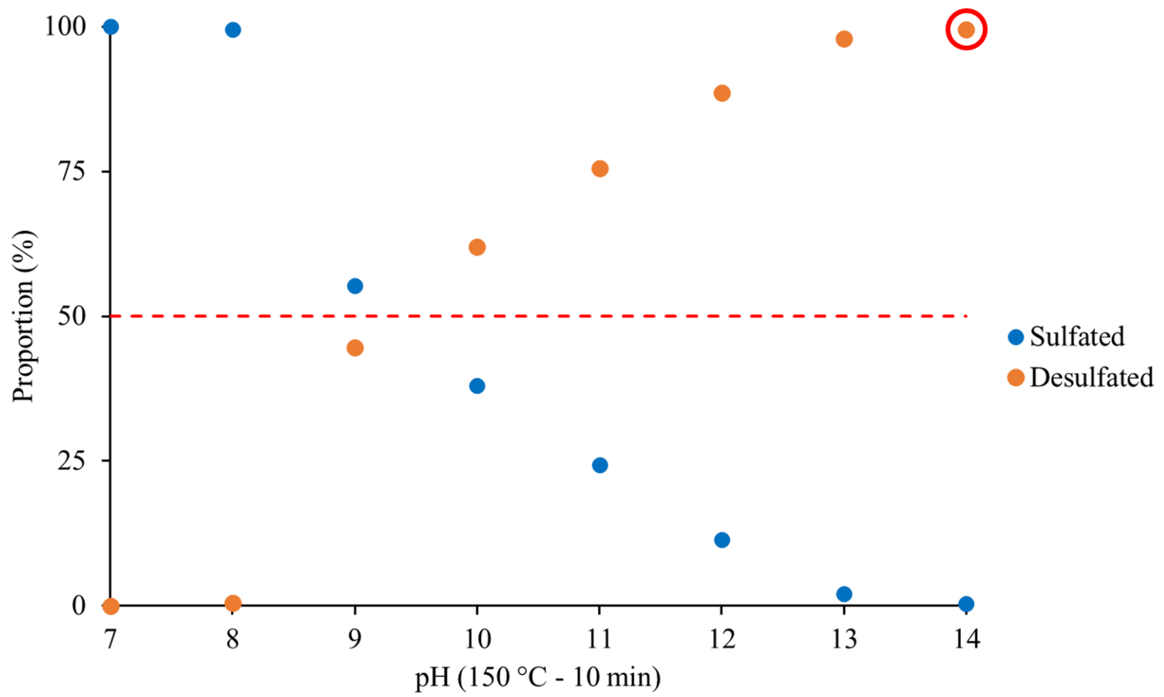

Figure S9: Microwave-assisted desulfation of *Holothuria scabra* sulfated saponins (150 °C for 10 min): influence of the pH (7 to 14) on the desulfation reactions as estimated by LC-MS(+)

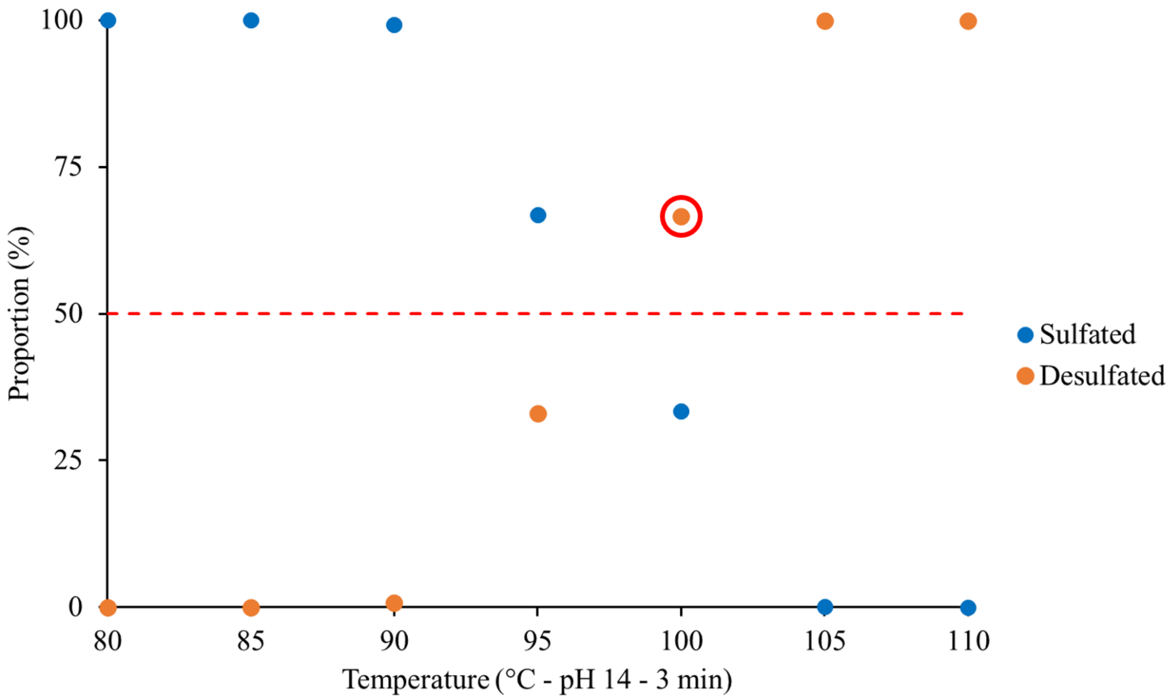

Figure S10: Microwave-assisted desulfation of *Holothuria scabra* sulfated saponins (pH 14 for 3 min): influence of the temperature (80 to 110 °C) on the desulfation reactions as estimated by LC-MS(+)

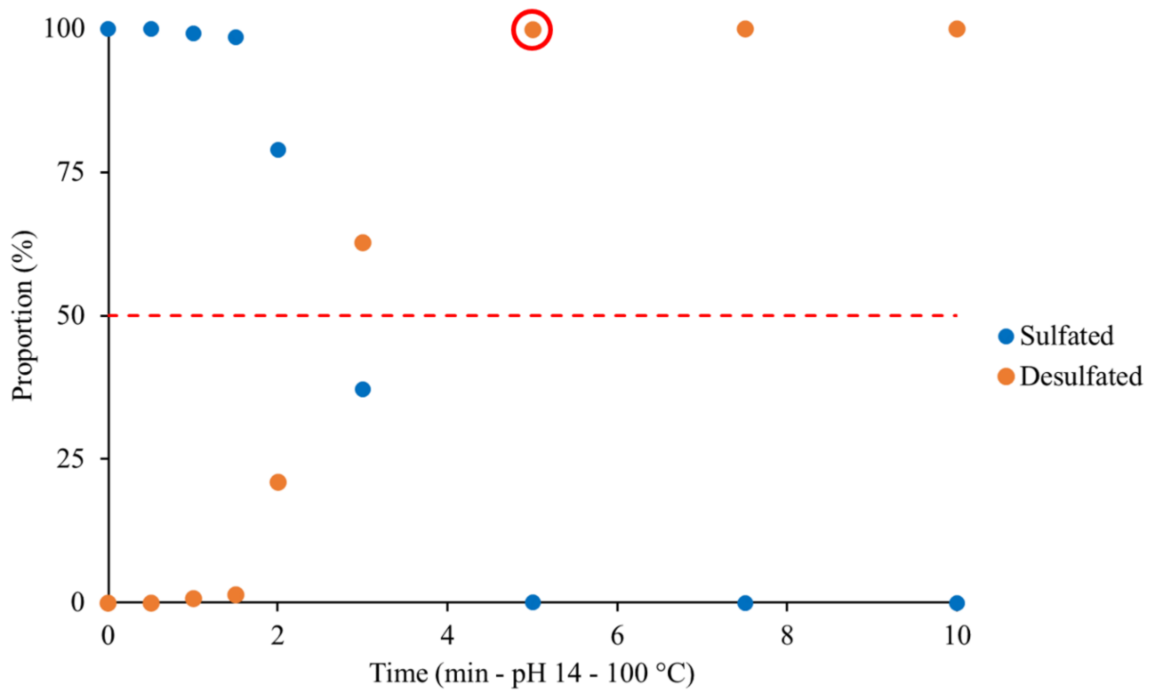

Figure S11: Microwave-assisted desulfation of *Holothuria scabra* sulfated saponins (pH 14 at 100 °C): influence of the time (1 s to 10 min) on the desulfation reactions as estimated by LC-MS(+)
